# Design and Experimental Validation of Pipeline Defect Detection in Low-Illumination Environments Based on Bionic Visual Perception

**DOI:** 10.3390/biomimetics10090569

**Published:** 2025-08-26

**Authors:** Xuan Xiao, Mingming Su, Bailiang Guo, Jingxue Wu, Jianming Wang, Jiayu Liang

**Affiliations:** 1 School of Computer Science and Technology, Tiangong University, Tianjin 300387, China; xxiao@tiangong.edu.cn (X.X.); wujingxue1014@163.com (J.W.); 2 School of Software, Tiangong University, Tianjin 300387, China; 2331111311@tiangong.edu.cn; 3 School of Electronics and Information Engineering, Tiangong University, Tianjin 300387, China; guobailiang@outlook.com; 4 Tianjin Key Laboratory of Autonomous Intelligence Technology and Systems, Tiangong University, Tianjin 300387, China; wangjianming@tiangong.edu.cn

**Keywords:** bio-inspired event vision, ES-YOLO, deep learning with Event data, synthetic event data, hyper-redundant manipulator, NMPC

## Abstract

Detecting internal defects in narrow and curved pipelines remains a significant challenge, due to the difficulty of achieving reliable defect perception under low-light conditions and generating collision-free motion trajectories. To address these challenges, this article proposes an event-aware ES-YOLO framework, and develops a pipeline defect inspection experimental environment that utilizes a hyper-redundant manipulator (HRM) to insert an event camera into the pipeline in a collision-free manner for defect inspection. First, to address the lack of datasets for event-based pipeline inspection, the ES-YOLO framework is proposed. This framework converts RGB data into an event dataset, N-neudet, which is subsequently used to train and evaluate the detection model. Concurrently, comparative experiments are conducted on steel and acrylic pipelines under three different illumination conditions. The experimental results demonstrate that, under low-light conditions, the event-based detection model significantly outperforms the RGB detection model in defect recognition rates for both types of pipelines. Second, a pipeline defect detection physical system is developed, integrating a visual perception module based on the ES-YOLO framework and a control module for the snake-like HRM. The system controls the HRM using a combination of Nonlinear Model Predictive Control (NMPC) and the Serpentine Crawling Algorithm (SCA), enabling the event camera to perform collision-free inspection within the pipeline. Finally, extensive pipeline insertion experiments are conducted to validate the feasibility and effectiveness of the proposed framework. The results demonstrate that the framework can effectively identify steel pipeline defects in a 2 Lux low-light environment, achieving a detection accuracy of 84%.

## 1. Introduction

Pipelines typically present a narrow, complex, and poorly illuminated environment [1], making manual inspection of pipeline defects a considerable challenge. This challenge arises primarily from two factors. First, inadequate lighting conditions hinder sensors from clearly capturing the internal pipeline environment. Second, the narrow and curved geometry of pipelines restricts the effectiveness of traditional manual inspection and remotely operated robotic systems, which are not only inefficient and vulnerable to environmental disturbances but also struggle to detect concealed defects. In recent years, with advancements in sensor technologies and intelligent robotics, pipeline inspection has increasingly evolved toward automation and intelligence.

Visual perception is widely applied in pipeline defect detection. Le et al. [2] developed an online multi-sensor inspection system for pipeline defect detection, employing RGB fusion technology to precisely identify defects such as cracks, corrosion, and blockages. Nevertheless, the limitations of RGB cameras in low-light and high-dynamic-range environments remain a significant challenge in complex pipeline inspections.

In recent years, event cameras, as bio-inspired visual sensors, have become increasingly important in industrial inspection due to their advantages in dynamic range, temporal resolution, low power consumption, and high sensitivity to low-light conditions [3]. This makes event cameras particularly suited for low-light and high-contrast environments. However, existing computer vision algorithms are not directly compatible with event streams, posing challenges for the direct application of event cameras in data processing. In addition, the data format of RGB and event streams differs significantly, and most existing standard datasets are based on RGB images, with a lack of publicly available datasets specifically designed for event data. This further restricts the application and development of event cameras in specific fields. Recreating pipeline defect datasets suitable for event cameras is not only time-consuming but also costly.

To address this issue, Henri Rebecq et al. [4] designed the event camera simulator ESIM. Building on this, Daniel Gehrig et al. [3] introduced frame upsampling technology into this framework, allowing models trained on synthetic event data to better generalize to real event data. Although these methods offer potential for applying event cameras to pipeline defect detection, whether the model can preserve the advantages of event cameras in low-light conditions remains a critical issue. Furthermore, beyond visual perception, ensuring that a pipeline inspection robot can navigate a collision-free trajectory for safe insertion into the pipeline constitutes an additional significant challenge. Currently, pipeline robots have been developed in various structures and configurations [5,6,7]. These technologies offer significant advantages in improving detection accuracy and reducing labor costs.

Among them, snake-like hyper-redundant manipulators (HRMs), due to their high flexibility, adaptability, and multiple degrees of freedom, can operate efficiently in complex pipeline environments. However, a key challenge in their path planning lies in ensuring that the end-effector reaches the target position while avoiding collisions between the manipulator and its surroundings. Although the high redundancy enhances the manipulator’s reachability and spatial exploration capabilities, it also significantly increases the computational time and resource consumption of path planning [8,9]. The E-RRT* algorithm proposed by Ji et al. [10] employed ellipses instead of traditional straight lines to connect path nodes and optimized the sampling process, effectively addressing the path planning problem for HRMs in confined spaces.

Due to their slender and flexible bodies, snakes efficiently explore narrow spaces, making them a key inspiration for bionic design. Inspired by this, previous studies have proposed modular snake-like structures to construct HRMs [11]. Furthermore, snake locomotion exhibits a “head-following” characteristic, whereby body movements are determined by the trajectory of the head. This mechanism can be applied to the motion planning of snake-like robots: if the final configuration of the robot’s end-effector reaching the target point is directly planned, this configuration can be regarded as the head trajectory, and the body joints follow this trajectory. Zhu et al. [12] proposed a pipeline inspection method that combines Nonlinear Model Predictive Control (NMPC) with a Serpentine-inspired Crawling Algorithm (SCA).

**Figure 1:**
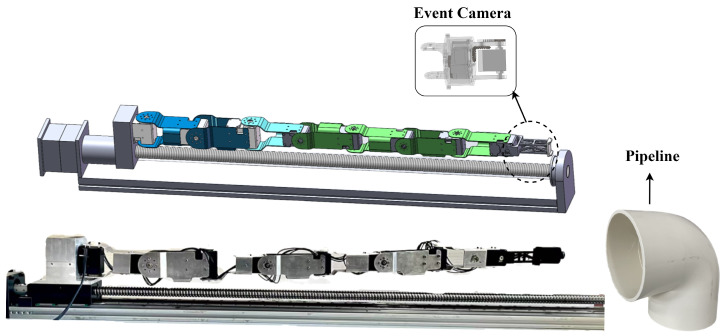
Schematic diagram of the model of an HRM equipped with an event camera.

In summary, this article proposes and validates an innovative framework, ES-YOLO, designed to address the core challenges of employing event cameras for defect detection in narrow, low-light pipelines. To this end, we first present a practical method to systematically convert a public RGB dataset (NEU-DET) into event-based data, thereby creating a novel dataset, N-neudet. This dataset is then utilized to train an efficient defect detection model. Furthermore, the proposed perception framework is integrated with the motion control system of a snake-like hyper-redundant manipulator, as illustrated in Figure 1. The feasibility and practical effectiveness of the overall system are validated through physical experiments conducted in pipelines of different materials and under extremely low-light conditions. The main contributions of this article are as follows.

- To the best knowledge of the authors, this work presents the first system-level integration and validation of event-driven perception with an HRM for pipeline inspection, thereby constructing and experimentally verifying a complete HRM-based defect detection platform. The contribution goes beyond algorithmic testing under idealized conditions by providing a holistic validation of the entire perception and motion framework under real-world physical constraints. These results convincingly demonstrate both the feasibility and the significant potential of this technical approach for addressing complex industrial environments.
- An event-based defect dataset, termed N-neudet, is successfully constructed, with its data synthesized from traditional RGB images using the ES-YOLO framework. The practical utility of this dataset is rigorously validated through comprehensive comparative experiments. A defect detection system trained on N-neudet exhibits remarkable stability and accuracy, particularly under low-light conditions where conventional RGB cameras proved ineffective. These results confirm the robustness of the dataset and its suitability for event-based pipeline defect detection applications.

## 2. Related Work

### 2.1. Trajectory Planning for HRM

Due to their high redundancy, HRMs can flexibly avoid obstacles and effectively access narrow, curved spaces.

The nonlinear control of HRMs poses significant challenges due to their high degrees of redundancy. Liu et al. [13] addressed this issue by proposing a novel control framework based on a nonlinear observer, which transformed the system into an interconnected input-to-state stable (ISS) structure, thereby achieving asymptotic stability. Building upon this, Liu et al. [14] employed the small-gain theorem to design distributed optimal controllers that effectively resolved the output consensus problem in multi-agent nonlinear systems. More recently, Jin [15] tackled the global asymptotic stability problem of feedback optimization in nonlinear systems by integrating an enhanced gradient flow optimizer with a nonlinear perturbation function. The proposed method was rigorously validated using tools such as singular perturbation theory and input-to-state stability analysis.These studies provide theoretical guidance and inspiration for the nonlinear control of HRMs.

Currently, prevalent path planning methods primarily include graph search-based algorithms and sampling-based algorithms. Among these, the A* algorithm [16,17,18] and Dijkstra’s algorithm [9] were common representatives of graph search methods. Tang et al. [19] enhanced the A* algorithm by incorporating the artificial potential field method, introducing new node search strategies, and integrating local path optimization. This approach effectively reduced the number of search nodes, improved search efficiency, and simultaneously optimized the manipulator’s obstacle avoidance posture. Conversely, ref. [20] proposed an extended Dijkstra’s algorithm that integrates Delaunay triangulation and plane transformation techniques to optimize paths on complex surfaces, significantly enhancing path planning accuracy in both single-robot and multi-robot tasks.

Ref. [12] proposed a pipeline inspection and detection method that integrates Nonlinear Model Predictive Control (NMPC) with a Snake-inspired Heuristic Crawling Algorithm (SCA). The method is divided into three stages: insertion, inspection, and exit. During the insertion and withdrawal stages, the SCA—inspired by snake locomotion—is employed to significantly reduce path planning time. In the inspection stage, NMPC is applied to ensure efficient and collision-free configuration. Specifically, NMPC functions as a high-level planner, generating a global, collision-free path configuration. In contrast, SCA operates as a low-level controller, guiding the manipulator to accurately track the sequence of path points generated by NMPC, thereby executing the physical insertion motion.

### 2.2. Deep Learning with Event Data

In the field of event cameras, significant efforts have been made to create datasets and explore their applications, particularly in object detection.

For example, in [21], the authors used a Spiking Neural Network (SNN) to simulate human eye saccadic movements, converting static image datasets into event data, thereby creating semi-synthetic event-based versions of the MNIST and Caltech101 datasets. In [22], the DVS-Gesture dataset, which includes 11 types of gestures, was used to achieve real-time recognition of gesture data streamed by DVS. Additionally, Enrico Calabrese et al. [23] created a 3D human pose dataset, and [24] created the active pixel vision sensors (DAVIS) Driving Dataset (DDD17), the first driving recording dataset combining DVS and DAVIS. Subsequently, ref. [25] proposed DSEC, a large-scale new dataset that includes data from high-resolution event cameras, traditional cameras, LiDAR, and GPS, and provides disparity ground truth, aimed at promoting and evaluating the development of event-based stereo vision algorithms.

These datasets have laid the foundation for advancements in object detection using event cameras. Cannici et al. [26] proposed two event camera-based object detection models, YOLE and fcYOLE. Maqueda et al. [27] designed a CNN architecture adapted to event camera outputs and proposed an event-frame representation method, successfully predicting steering angles in autonomous driving using the DDD17 [24] dataset based on event cameras. Subsequently, Alonso et al. [28] proposed the first semantic segmentation baseline model for the DDD17 dataset. However, event-based deep learning algorithms still lack support from large-scale event datasets.

### 2.3. Synthetic Event Data

Currently, there are many event datasets [21,22,23] available for event-driven algorithms. Rebecq et al. [29,30] made progress by using simulated event data to train a recurrent neural network-based model, which succeeded in video reconstruction tasks, demonstrating the potential for expanding event-camera applications. Kaiser et al. [31] proposed a simple event camera simulator that generates events based on image difference thresholds. Refs. [32,33] introduced two event generation simulators. Daniel Gehrig et al. [3] enhanced the event simulator ESIM [4] with frame interpolation technology, which can convert frame data from traditional video sequences into asynchronous event streams. This method allows for the reuse of existing datasets to generate event data and accurately simulates the behavior of real event cameras, ensuring that synthetic data is suitable for training and testing event algorithms.

## 3. Overall Workflow of the Pipeline Defect Detection Method

To evaluate a performance of the ES-YOLO framework in a real-world pipeline environment, a dedicated experimental platform for defect detection is developed. This platform integrates two core components: first, a visual perception module equipped with the ES-YOLO framework to address the challenge of detection in low-light environments; second, a snake-like HRM motion control system based on NMPC and SCA algorithms to achieve collision-free navigation within complex curved pipelines.

The experimental procedure is primarily divided into three steps.

- Step 1: Model Construction. The ESIM model is employed to synthesize an event-based dataset, designated N-neudet, from the public NEU-DET RGB dataset. This synthesized dataset is then utilized to train a YOLOv8 model, culminating in the final ES-YOLO framework.
- Step 2: Data Collection. An event camera is mounted on the HRM. Guided by the NMPC and SCA algorithms, the HRM first determines a collision-free trajectory and is subsequently inserted into the pipeline to collect in situ data.
- Step 3: Defect Detection and Evaluation. Upon completion of data acquisition, the resulting dataset is fed into the ES-YOLO framework for defect detection, and the performance is quantitatively evaluated.

**Figure 2:**
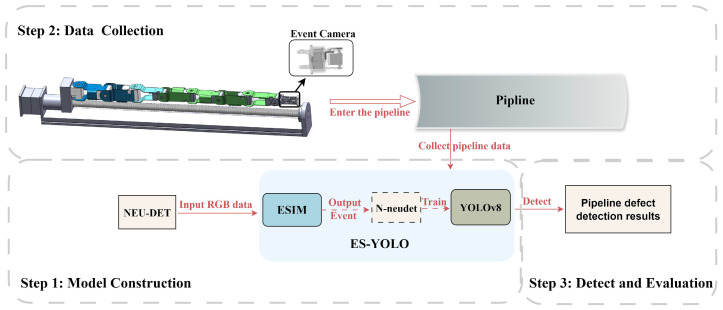
Overall workflow of the pipeline defect detection method based on the ES-YOLO.

The comprehensive experimental workflow is illustrated in Figure 2.

## 4. Methodology of the ES-YOLO

### 4.1. Overview

**Figure 3:**
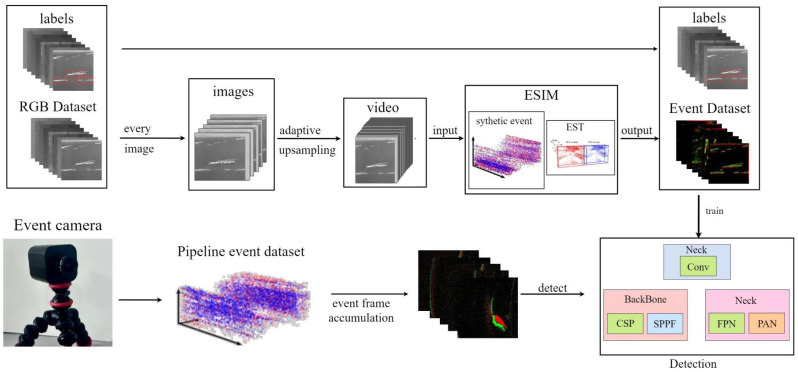
ES-YOLO framework architecture. First, large-scale image datasets (NEU-DET) are converted into synthetic event datasets (N-neudet) through the ESIM module; then, the generated event data is passed to the YOLOv8 module for training.

The framework of ES-YOLO, which converts RGB datasets for event cameras, is illustrated in Figure 3.

ES-YOLO consists of the ESIM module and the YOLOv8s module. This section introduces the ESIM module and the YOLOv8s module. First, the RGB dataset is upsampled to convert it into a high-frequency dataset. Second, the ESIM module is used to synthesize event dataset. Finally, the YOLOv8s model is trained using the N-neudet event dataset, and defect detection is performed on actual pipelines.

### 4.2. ESIM Module

ESIM module is used to synthesize event data. Due to the lack of large event datasets for defect detection, we need to convert RGB datasets into event datasets. Equation (1) [3,34,35] describes the event generation model of an ideal sensor. The pixels of an event camera are independent and continuously monitor the logarithmic brightness signal at their corresponding positions L(u,t). When the change in logarithmic brightness $u={\left(x_{i},y_{i}\right)}^{T}$ at a pixel exceeds a certain threshold *C* over time $t_{i}$, an event $e_{i}=\left(x_{i},y_{i},t_{i},p_{i}\right)$ is triggered.

(1) $$\Delta L\left({\left(x_{i},y_{i}\right)}^{T},t_{i}\right)=L\left({\left(x_{i},y_{i}\right)}^{T},t_{i}\right)-L\left({\left(x_{i},y_{i}\right)}^{T},t_{i}-\Delta t_{i}\right)\geq p_{i}C$$

In Equation (1), $p_{i}\in \left\{-1,1\right\}$ represents the polarity of the event signal, which is the indicator of the change in brightness. Δt represents the time since the last occurrence of pixel *u*.

The image is processed through an offset function to transform the original image into a low-frame-rate version. Although the ESIM event camera simulator can adaptively render virtual scenes at any temporal resolution, the actual event camera operates on a microsecond timescale. Therefore, we apply frame interpolation technology [36] to enable frame reconstruction at any temporal resolution. Simultaneously, an adaptive upsampling strategy [4] is employed to determine the number of intermediate frames to generate, thus converting the low-frame-rate images into a high-frame-rate sequence. Finally, the event synthesis module (ESIM) is used to convert the high-frame-rate image sequence into event data.

### 4.3. YOLOv8 Module

The event dataset generated by ESIM in Section 4.2 is then used as input for training the defect detection model. As mentioned in [37], YOLO is the first model to treat the object detection problem as a regression task. Released in 2023, YOLOv8 [38] builds upon the success of earlier versions such as YOLOv5 [37], introducing advanced architectural designs and training strategies, including anchor-free detection, to provide a unified framework with improved accuracy for various computer vision tasks [39,40]. By integrating an anchor-free design with attention mechanisms and dynamic convolution, the model not only simplifies its architecture but also significantly enhances the detection of small objects—an essential factor in many edge deployment scenarios. Given its excellent performance in terms of accuracy, efficiency, and usability, this study adopts YOLOv8 as the core detection module.

### 4.4. Event Spike Tensor

The synthetic events are combined with the original NEU-DET labels to train the YOLO module. Due to the sparsity of event signals and their non-uniform spatiotemporal distribution, pattern recognition algorithms typically aggregate event data into grid-based representations. To address this, we use the general framework Event Spike Tensor (EST) [41], which converts event streams into grid-based representations. Unlike previous event representation methods, EST retains the four dimensions of event signals and maps events of positive and negative polarities to two independent spatiotemporal grids, thereby enhancing the model’s ability to represent event data.

## 5. Comparative Experiments Based on the NEU-DET Dataset

To validate the feasibility of the ES-YOLO framework for practical pipeline defect detection, we conducted a series of experiments. These experiments involved synthesizing event datasets, setting model training parameters, evaluating the model, and performing practical defect detection. Finally, we compared the detection results of the event camera with those of the RGB camera.

### 5.1. Dataset Generation

**Figure 4:**
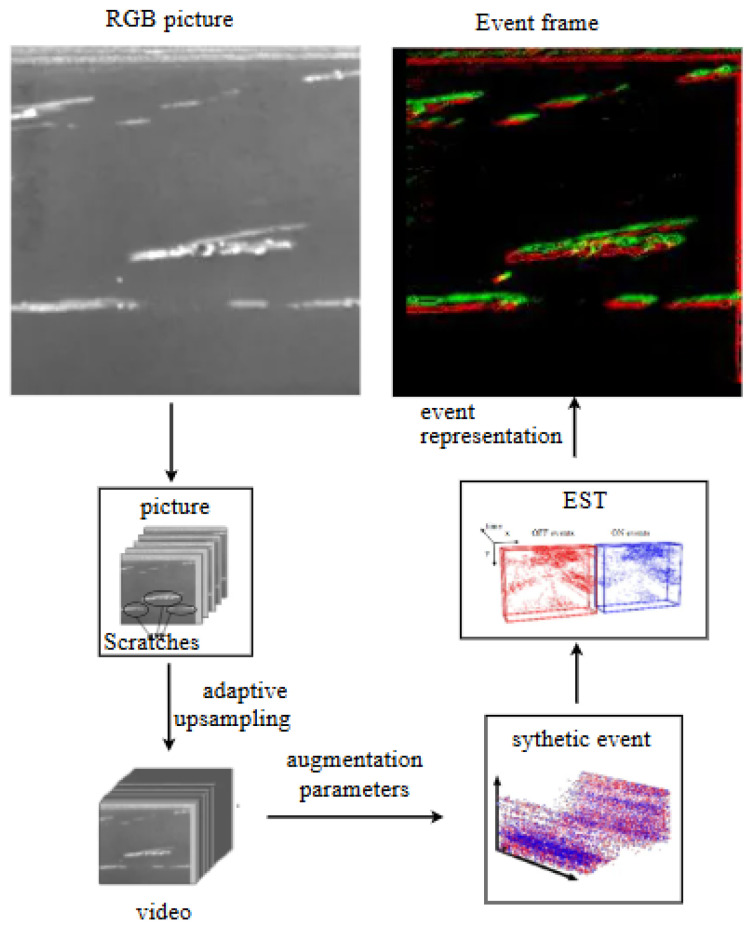
Illustration of RGB dataset conversed into event dataset.

For the experiments, we selected the NEU-DET dataset [42], which contained six typical surface defects of hot-rolled strip steel: Roll Scale (RS), Patches (Pa), Cracks (Cr), Pitted Surface (PS), Inclusions (In), and Scratches (Sc). The dataset consisted of 1800 images, with 300 samples per defect type, and included detailed annotations specifying both the defect categories and their locations. After selecting the NEU-DET dataset, we processed it through the ESIM module to generate a synthetic event-based defect dataset, which we named N-neudet. This event dataset was subsequently used to train the YOLO-based defect detection model, allowing for the evaluation of the model’s performance across different defect categories. As a result, a total of 1800 event defect images were generated, containing six different types of typical defects, with 300 samples for each defect type. The conversion process from RGB images to event frames is illustrated in Figure 4.

To improve the recognition accuracy, this article employed a sliding window combined with adaptive interpolation strategy. Specifically, a 512 × 512 Region of Interest (ROI) window was defined on a 640 × 640 static RGB image and was synchronously shifted in both horizontal and vertical directions with a stride of s=1 pixel, resulting in $K=\left\lceil \frac{640-512}{s}\right\rceil +1=129$ sub-regions (with the final incomplete region discarded). Each ROI sequence was synthesized into a 128-frame, 20 fps video, while its original label boundaries were mapped to the local coordinate system. This transformation was given by $x^{\prime }=x-\Delta x$, where x represented the bounding box coordinates in the original image, Δx was the displacement vector of the ROI window’s origin, and $x^{\prime }$ was the resulting coordinate vector in the local ROI frame. Subsequently, an adaptive frame interpolation technique [36] was applied to upsample the video from 20 fps to 200 fps, producing a high-frame-rate sequence. This sequence was then fed into the ESIM module to generate the corresponding event data. Compared to directly simulating events from the full 640 × 640 image, the proposed sliding window and adaptive interpolation approach significantly increased the target event density, thereby enhancing the recognition accuracy of the ES-YOLO framework.

To simulate event generation, we used the open-source simulator ESIM [3]. Before generating events, we needed to carefully select the contrast thresholds. We randomly sampled the contrast thresholds for positive ($C_{tp}$) and negative ($C_{tn}$) events from a uniform distribution, $U(C_{t\min},C_{t\max})$. This random sampling method improved the domain adaptability between simulated and real data, thereby enhancing the generalization of simulated events to real events. In this experiment, we chose $C_{t\min}=0.1$ and $C_{t\max}=0.5$. After the ESIM simulation, the final event stream was generated with an event density of 256 events/ms and a temporal resolution of 1.953 ms.

Finally, to match the synthesized event stream with the annotations based on the YOLOv8 model, we converted the asynchronous and sparse event stream into a tensor representation. We selected the Event Spike Tensor (EST) [41] because it performed well in both high-level and low-level tasks. The EST method mapped positive and negative polarity events into separate spatiotemporal grids, which were then stacked along the channel dimension to form the final tensor representation of size H×W×C. In this representation, *H* and *W* were the spatial resolution of the sensor, and C=15 was a hyperparameter which controls the number of temporal bins used to aggregate events.

### 5.2. Experimental Parameters

This experiment was implemented using the PyTorch framework. The ADAM optimizer was used for training, with a learning rate set to 0.01. A total of 100 epochs were trained, with the momentum parameter set to 0.937, and a batch size of 64. The training parameters used in the experiment are shown in Table 1.

### 5.3. Evaluation Metrics

In this experiment, we used multiple metrics to evaluate the model’s detection performance, including precision (*P*), recall (*R*), and mean average precision (mAP).

Precision is defined as the ratio of the number of True Positive samples correctly predicted by the model to the total number of samples predicted as positive by the model, as shown in Equation (2).

(2) $$\mathrm{Precision}=\frac{\mathrm{TP}}{\mathrm{TP}+\mathrm{FP}}$$

Recall is defined as the ratio of the number of True Positive samples correctly predicted by the model to the total number of actual True Positive samples, as shown in the following Equation (3).

(3) $$\mathrm{Recall}=\frac{\mathrm{TP}}{\mathrm{TP}+\mathrm{FN}}$$

mAP and AP are metrics used to evaluate multi-class classification problems. mAP is the average of the AP values across all classes, while AP is calculated separately for each class. The formulas are shown in Equations (4) and (5).

(4) $$\mathrm{AP}=\int_{0}^{1}P\left(R\right)\;dR$$

(5) $$\mathrm{mAP}=\frac{\sum_{j=1}^{S}\mathrm{AP}\left(j\right)}{S}$$

In the above formula, *S* represents the total number of classes, TP is True Positives, FP is False Positives, and FN is False Negatives. P(R) is the precision–recall curve. These metrics are important for evaluating the performance and adaptability of the model.

The False Negative Rate (FNR) is defined as the proportion of positive samples that the model fails to detect. It reflects the model’s missed detection rate, as shown in Equation (6).

(6) $$\mathrm{FNR}=\frac{\mathrm{FN}}{\mathrm{TP}+\mathrm{FN}}=1-\mathrm{Recall}$$

In traditional classification tasks, the False Positive Rate (FPR), defined as the proportion of non-defective samples incorrectly classified as defective, is commonly used to evaluate model performance. However, in the context of industrial defect inspection, this metric may not provide a meaningful assessment of model reliability. To more effectively capture the practical impact of false alarms in such scenarios, this study adopts the False Discovery Rate (FDR) as a key evaluation metric. FDR is defined as the proportion of false positives among all samples predicted as defective, thereby directly quantifying the severity of the model’s false alarms. The formulation of FDR is presented in Equation (7).

(7) $$\mathrm{FDR}=\frac{\mathrm{FP}}{\mathrm{TP}+\mathrm{FP}}=1-\mathrm{Precision}$$

### 5.4. Results

#### 5.4.1. Training Visualization

**Figure 5:**
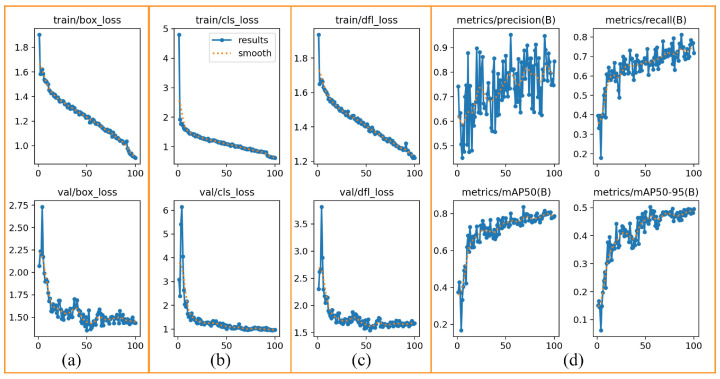
Training result figures. (**a**) The bounding box loss (box_loss), which measures the error in predicted object locations, shows a consistent downward trend, indicating that the model is effectively learning to locate objects; (**b**) the classification loss (cls_loss), indicating the error in predicting the correct object class, also steadily decreases as training progresses; (**c**) Distribution Focal Loss (dfl_loss), another component for bounding box regression, follows a similar convergence pattern; (**d**) Key evaluation metrics, showing the progression of precision, recall, and mean Average Precision (mAP), all exhibit a clear upward trend, signifying significant performance improvement over time.

Through the experiments described above, we obtained the ES-YOLO framework’s experimental results on the N-neudet training set, as illustrated in Figure 5.

In Figure 5a, the train/box_loss exhibited a steady downward trend, indicating that the model’s bounding box regression capability was gradually improved. The val/box_loss also showed a similar downward trend, confirming the model’s strong generalization ability on unseen data. In Figure 5b, both train/cls_loss and val/cls_loss decreased significantly, signifying that the model was effectively learning to distinguish between different object classes. The distribution focal loss, another component of bounding box regression, was shown in Figure 5c. Its convergence on both sets further confirmed the stability of the training process.

In Figure 5d, as the number of training epochs increased, the model’s precision gradually improved. The mAP@0.5 (mean average precision at an IoU threshold of 0.5) increased rapidly and stabilized, indicating a significant enhancement in the model’s overall detection performance. Although the mAP@0.5:0.95 (mean average precision across IoU thresholds from 0.5 to 0.95) was lower than mAP@0.5, its upward trend was also evident, further demonstrating the model’s robustness across different IoU thresholds.

Overall, the ES-YOLO framework exhibited good convergence during training, with both training and validation losses showing a downward trend. Additionally, the model’s precision and recall demonstrated significant improvement, highlighting the model’s effectiveness in the defect detection task.

#### 5.4.2. Validating Visualization

We selected a subset of images from the N-neudet event dataset as the validation set. After 100 epochs of training, the model’s performance on the validation set is presented in Table 2.

The results showed that the model achieved a precision of 0.952, a recall of 0.657, and an mAP@0.5 of 0.801 across all six categories, demonstrating good overall performance. However, for the two defect types, Crazing and Rolled-in-scale, although the detection precision exceeded 0.8, the recall rates were notably low. This phenomenon indicated that the model struggled to accurately distinguish between these two specific defect types, often leading to misclassifications. These findings highlighted certain limitations of event cameras in fine-grained defect classification tasks.

**Figure 6:**
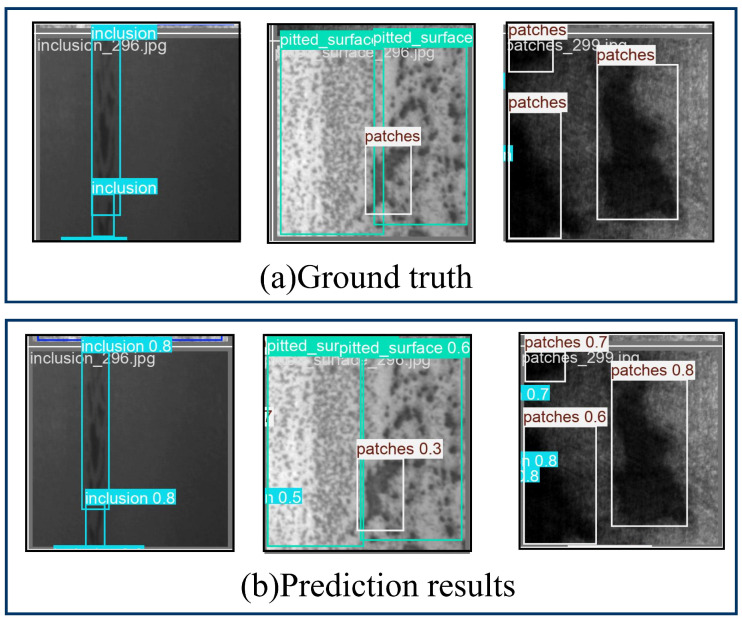
Visualization of validation set results. (**a**) represents the ground truth; (**b**) represents the prediction results of the validation set.

The qualitative results of the validation set are illustrated in Figure 6. The bounding boxes in Figure 6a represent the ground truth. Figure 6b illustrates the prediction results of the validation set.

Meanwhile, the original NEU-DET dataset was trained using the YOLOv8s model, and detection results were obtained on the validation set. Table 3 presents a quantitative comparison of the detection performance between models trained on the original NEU-DET RGB dataset and the synthesized N-NEUDET event dataset. The evaluation was conducted using the four key metrics of precision (P), recall (R), mAP@0.5, and inference time. The results demonstrated that, on the validation set, the ES-YOLO model achieved substantially higher detection accuracy on the event-based dataset compared to the RGB dataset, which validated its effectiveness in defect identification.

## 6. Performance Evaluation of ES-YOLO Under Different Illuminations

To validate the advantages of event cameras in defect detection, we conducted comparative experiments. Specifically, we used the Intel RealSense L515 RGB camera and the DVXplorer Mini event camera to collect data from steel and acrylic pipelines. The trained ES-YOLO framework was then applied for detection, and the results were analyzed for comparison.

**Figure 7:**
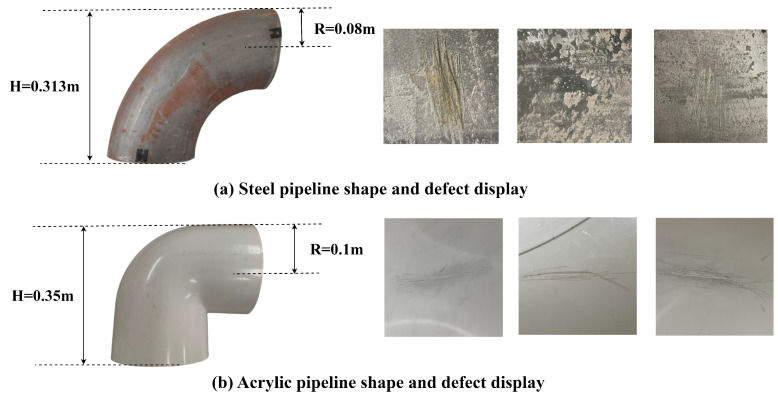
Diagram showing the radius height of the experimental pipeline and its defects.

The geometric specifications and defect characteristics of the two pipeline types are depicted in Figure 7. Figure 7a presents the steel pipeline, which was sourced from a decommissioned industrial system and contained naturally occurring defects such as rust patches, surface scratches, and pitting corrosion. In contrast, Figure 7b displays the acrylic pipe, on which scratches were artificially created to simulate common damage, given its material properties. In this experiment, we used the detection rate ($D_{r}$) metric to evaluate the performance of the ES-YOLO framework. The detection rate was defined as the ratio of the number of defect samples successfully detected by the framework to the total number of defect samples captured by the camera, as expressed in Equation (8).

(8) $$D_{r}=\frac{D_{s}}{D_{s}+D_{f}}$$

where $D_{s}$ represents the number of defect samples successfully detected, and $D_{f}$ represents the number of defect samples not detected.

**Figure 8:**
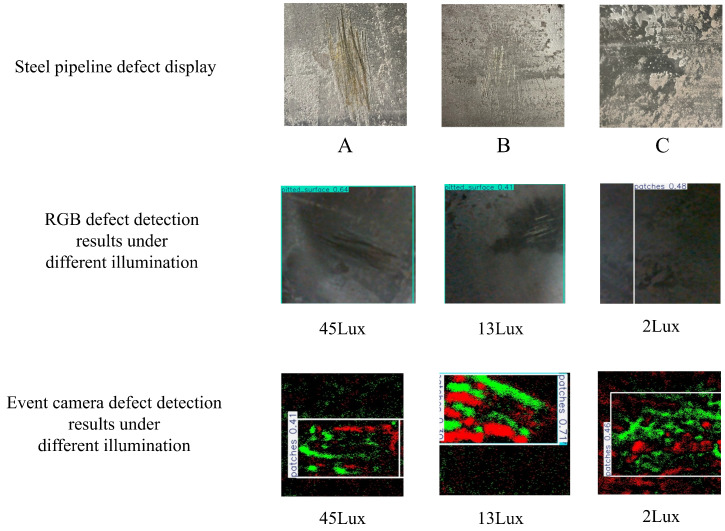
Comparison experiment of different defects and illumination levels in steel pipes. Among them, A, B, and C represent the target defects in the steel pipe. Both the event camera and RGB camera can detect the defect at all three points (A, B, and C), where the illumination levels are 45 Lux, 13 Lux, and 2 Lux, respectively.

**Figure 9:**
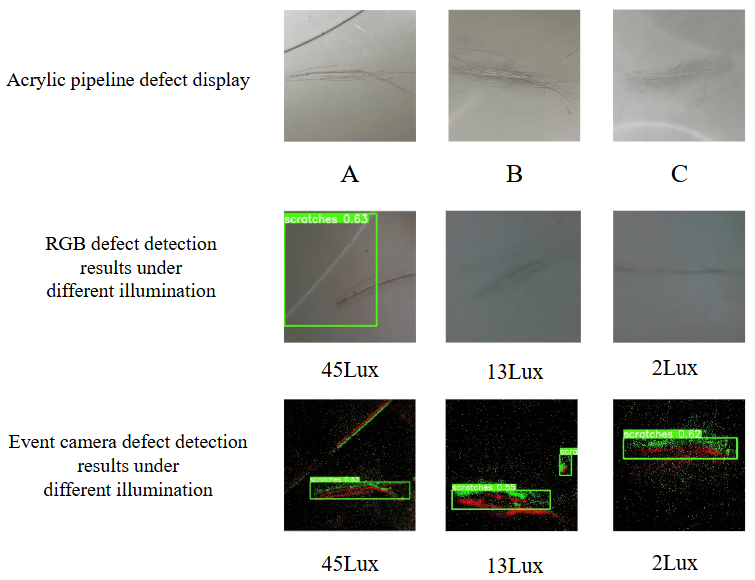
Comparison experiment of different defects and illumination levels in acrylic pipes. Among them, A, B, and C represent the target defects in the acrylic pipe. The illumination levels are 13 Lux and 2 Lux when measured at points B and C, respectively. As a result, only the event camera successfully detected the defects.

In the actual pipeline inspection experiment, we designed six comparative experiments to evaluate the detection performance of event cameras and RGB cameras under the same target defects and lighting conditions. First, three target defects were selected on both steel and acrylic pipelines, with their specific locations marked as A, B, and C in Figure 8 and Figure 9. Then, we used an optical illuminance meter UT383 to measure the brightness of the target points, where the illuminance of defects A, B, and C was 45 Lux, 13 Lux, and 2 Lux, respectively. Subsequently, both event and RGB cameras were used to capture images of the target defects on both types of pipelines. A total of 50 images were collected for each target defect using the Intel RealSense L515 RGB camera and the DVXplorer Mini event-based camera, respectively. The collected images were then fed into the ES-YOLO framework for detection.

Figure 8 and Figure 9 present the defect images collected from the steel and acrylic pipelines, respectively. To facilitate a direct comparison of the performance between the two sensors, these figures also include the corresponding detection results obtained from the RGB camera and the event camera under three distinct lighting conditions. Notably, in the low-light environment of 2 Lux, the RGB camera exhibited markedly inferior detection performance compared to the event camera, with frequent occurrences of misclassifications and even complete missed detections. The quantitative detection results for the steel and acrylic pipelines are detailed in Table 4 and Table 5, respectively.

The experimental results showed that in the steel pipeline, the event camera successfully detected all target defects, achieving a detection rate of over 84%. In contrast, the defect detection rate using RGB images under 2 Lux low-light conditions was only 10%, which was significantly lower than the detection performance achieved with event-based data. In the acrylic pipeline, the RGB camera detected defects only under 45 Lux and 13 Lux lighting conditions, with a maximum detection rate of 34%. In contrast, the event camera accurately detected the target defects under all lighting conditions, with a detection rate consistently above 60%. These results indicate that, compared to the RGB camera, the event camera could effectively detect pipeline defects in low-light environments. However, the detection rate of the RGB model for acrylic pipelines was significantly lower than that for steel pipelines, primarily due to the traditional RGB models’ susceptibility to interference caused by the optical properties of different materials. In contrast, the event camera effectively alleviated the impact of material differences by capturing the dynamic characteristics of defects. The results indicate that the ES-YOLO framework performed better in cross-material detection.

In summary, under the low-light environments, the event camera consistently performed better than the RGB camera, particularly in low-light and cross-material scenarios, which made it more suitable for practical applications and provided a significant overall advantage.

## 7. Experimental Design for Pipeline Defect Detection via a Snake-like HRM

### 7.1. Composition of the Pipeline Defect Detection System

**Figure 10:**
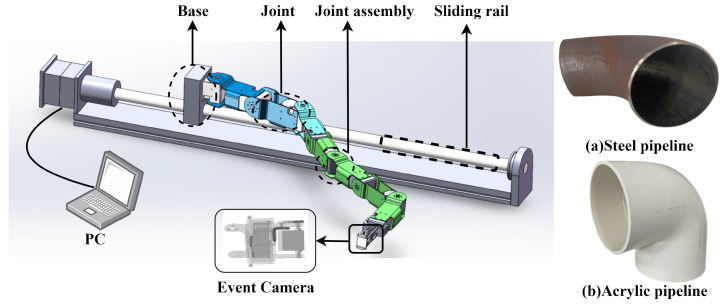
Schematic diagram of the pipeline defect detection system, where (**a**) represents the steel pipeline and (**b**) corresponds to the acrylic pipeline.

To evaluate the performance of the ES-YOLO framework in real-world pipeline environments, a pipeline defect detection experimental platform is constructed, comprising two main components. The first is a visual perception module based on the ES-YOLO framework, designed to tackle detection challenges under low-light conditions. The second is a modeling and control algorithm for the snake-like HRM, developed to enable collision-free navigation within curved pipeline structures. The system consists of an HRM, a sliding rail, a base, an event camera mounted on the end of the HRM, and a PC-based control system, as illustrated in Figure 10.

A key design consideration is achieving a balance between mechanical strength and maneuverability. The HRM adopts a “3 + 5” hierarchical configuration: three high-torque XH540-W270-R motors connected by magnesium–aluminum alloy joints form the base section, while five lightweight XH430-W270-R motors connected via resin connectors comprise the end section. The sliding rail structure employs a ball screw drive system, which consists primarily of a stepper motor, a lead screw, and a mounting base for the HRM. The ball screw system offers a travel distance of 1.1 m and features high load capacity, and stable stepping performance. The PC-based control system independently communicates control commands to both the HRM and the sliding rail using the RS485 protocol, ensuring reliable and synchronized motion control. Additionally, the DVXplorer Mini event camera is mounted on the HRM’s end-effector to capture pipeline defects throughout the insertion process. Table 6 shows more details of the system specifications.

**Figure 11:**
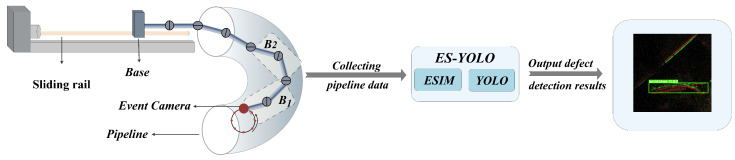
Schematic diagram of the pipeline defect detection experimental workflow.

The overall workflow of the system is illustrated in Figure 11. First, a control algorithm facilitates its collision-free insertion to accurately reach the target detection position. Subsequently, the event camera at the end of the HRM is used to collect internal data from within the pipeline. Finally, the acquired data is processed using the ES-YOLO framework, and defect detection results are produced.

### 7.2. The Model and Control Algorithm of the HRM

**Figure 12:**
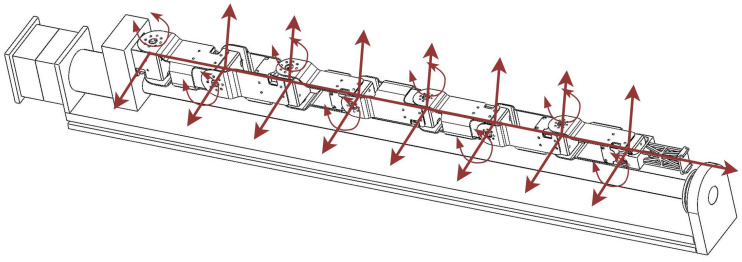
Mechanical structure design of HRM and sliding rail.

To enable collision-free defect inspection within narrow pipelines, this study employs a motor-driven, 9-degree-of-freedom (DOF) hyper-redundant snake-like robotic arm. The HRM consists of a base, a sliding rail, eight joints, and joint assemblies, with its detailed configuration illustrated in Figure 12.

To characterize the motion properties of the HRM, this article establishes its kinematic model based on forward kinematics and Denavit–Hartenberg (D-H) parameters. The relevant D-H parameters are presented in Table 7. Based on this model, the homogeneous transformation matrix Ti−1i(i=1,2…n) between adjacent joints is derived, as shown in Equation (9), where ‘s’ represents the sine function (sin), and ‘c’ represents the cosine function (cos).

(9) Ti−1i=cθi−sθicαisθisαiaicθisθicθicαi−cθisαiaisθi0sαicαidi0001

By combining the transformation matrix Tw0 from the world coordinate system to the base coordinate system and the homogeneous transformation matrix Twn from the world coordinate system to the end-effector, the forward kinematics equation is obtained, as shown in Equation (10). Here, $q=[\theta_{1},\theta_{2},...,\theta_{n}]$ denotes the configuration vector of the HRM.

(10) fkine(q)=Twn=Tw0T01…Tn−1n

**Figure 13:**
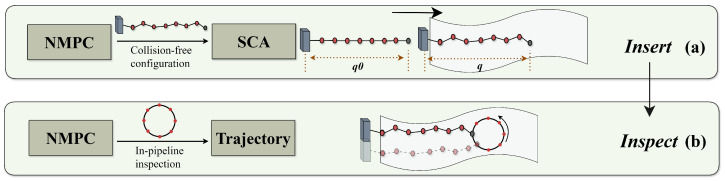
Flow chart of the pipeline inspection process: $q_{0}$ (initial horizontal configuration), q (collision-free configuration).

Due to the high degree of redundancy in the HRM and the spatial constraints of narrow pipeline environments, the control complexity of pipeline defect inspection tasks is significantly increased. To ensure collision-free operation of the HRM during inspection, efficient path planning is essential. However, due to kinematic constraints and the inherent complexity of the planning process, finding an optimal path often incurs substantial time and computational costs, and requires precise control of each HRM joint to avoid collisions. To balance planning efficiency and path quality, this article adopts an efficient strategy that seeks a suboptimal HRM configuration to reduce computational cost. This strategy is ultimately implemented by combining Nonlinear Model Predictive Control (NMPC) with a Snake-inspired Crawling Algorithm (SCA) [12]. The obstacle avoidance strategy for pipeline defect inspection includes two stages: insertion and inspection, as shown in Figure 13. Experimental validation demonstrates that the minimum pipeline the HRM can successfully traverse is a 90° curved pipe with an inner diameter of 8 cm and a length of 48 cm. This limit is jointly determined by the physical dimensions of the manipulator, the pipeline radius, and the joint bending capability.

### 7.3. Generalization Ability of NMPC

To achieve comprehensive data acquisition of the inner pipeline wall, the event camera mounted on the HRM’s end-effector performs a rotational scan along the pipeline cross-section. This scanning trajectory is discretized into $M_{i}\left(i=1,\ldots ,m\right)$ key target points. To ensure the HRM can safely and accurately reach each target point, the NMPC algorithm is employed to pre-plan a corresponding collision-free configuration, $q_{i}\in R^{n}$, for every target point before the manipulator enters the pipeline.

The optimization objective of this pose planning is to minimize the sum of the absolute values of the joint angles and the sum of the changes in joint angles between adjacent configurations. This objective helps prevent the HRM from moving to its joint limits and avoids abrupt movements caused by local minima. The planning method for the collision-free configuration of the *i*-th target point is shown in Equation (11), where $\theta_{ij}$ represents the *j*-th joint angle (j=1,…,n) of the *i*-th configuration; $x_{i}$ is the desired pose vector of the *i*-th target point; $\theta_{\mathrm{lb}}$, $\theta_{\mathrm{ub}}$ define the upper and lower limits of the HRM’s joint angles; and $D_{\mathrm{safe}}$ represents the minimum safe distance between the HRM and the pipeline.

(11) $$\begin{array}{c} \mathrm{minimize}\;\sum_{j=1}^{n}|\theta_{ij}|+\sum_{j=1}^{n}\left|\theta_{ij}-\theta_{i-1j}\right|\\ \mathrm{subject}\;\mathrm{to}\;\left\{\begin{array}{c} {∥x_{i}-\mathrm{pose}\left(\mathrm{fkine}\left(q_{i}\right)\right)∥}_{2}=0\\ D_{\mathrm{pipe}}^{j}⩽R-D_{\mathrm{safe}}\\ \theta_{ij}\in \left[\theta_{\mathrm{lb}},\theta_{\mathrm{ub}}\right]\\ ▵\theta_{\mathrm{lb}}⩽\left|\theta_{ij}-\theta_{i-1j}\right|⩽▵\theta_{\mathrm{ub}} \end{array}\right. \end{array}$$

A solution from the NMPC is not always guaranteed due to multiple constraints, including the desired pose of the HRM end-effector, pipeline curvature, radius, and the safe distance $D_{\mathrm{safe}}$. In the experiments, $D_{\mathrm{safe}}$ is set to 4 cm. This threshold accounts for both the physical radius of the HRM links and potential positioning errors during actual operation, thereby ensuring physical safety. Planning is considered to have failed under two conditions.

**Figure 14:**
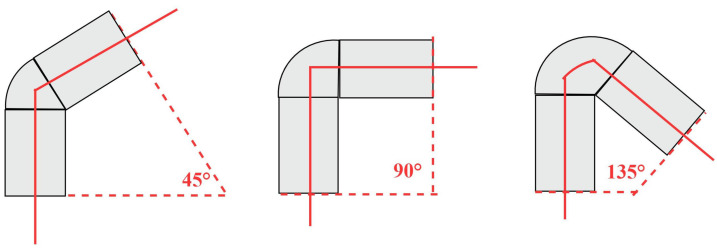
Schematic diagram of single-bend pipe models with different curvatures, where the red solid line represents the pipeline centerline.

To systematically evaluate the generalization ability of NMPC, this article conducts a series of collision-free configuration simulations in MATLAB. The objective of NMPC is to compute safe, collision-free configurations. For the HRM, configurations closer to the pipeline centerline reduce the risk of collision with the wall and thereby improve safety. Therefore, this simulation aims to calculate the maximum distance, $D_{\max}$, that the HRM’s configuration deviates from the centerline as it passes through pipelines of different curvatures. The minimum pipeline radius for ensuring safe passage is then calculated by adding this maximum deviation distance, $D_{\max}$, to a predefined safety threshold, $D_{\mathrm{safe}}$, where curvature is defined as the included angle of the sector forming the bent pipe section. The experiments test three typical pipeline curvatures, including $45^{\circ }$, $90^{\circ }$, $135^{\circ }$. The total length of all simulated pipelines is kept consistent, and their shapes are illustrated in Figure 14. Although the simulation results cannot directly quantify the algorithm’s generalization ability, they provide a qualitative evaluation of its adaptability under different geometric constraints. The specific experimental results are detailed in Table 8.

**Figure 15:**
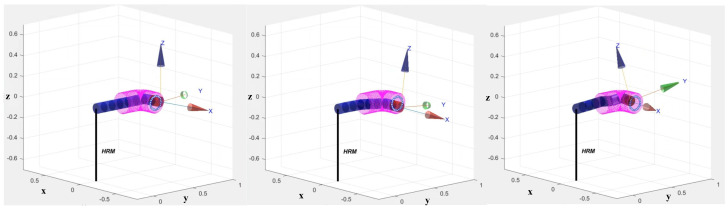
Simulation results for the collision-free inspection of the pipeline defect detection system.

The experimental results indicate that when the NMPC algorithm generates a collision-free configuration for a specific target point, higher pipeline curvature requires a larger radius to ensure safe passage. Specifically, for a pipeline with a $45^{\circ }$ curvature, the minimum passage radius required by the collision-free configuration planned by NMPC is 6.53 cm. When the curvature is $90^{\circ }$, the minimum pipeline radius the HRM can pass through is 7.72 cm. For a high-curvature pipeline of $135^{\circ }$, the pipeline radius must be at least 9.13 cm to ensure the HRM can perform the inspection safely. Figure 15 illustrates a successful planning result for a pipeline with a radius of 10 cm and a curvature of $90^{\circ }$, along with the corresponding collision-free configuration of the HRM.

## 8. Experimental Results for Pipeline Defect Detection via a Snake-like HRM

**Figure 16:**
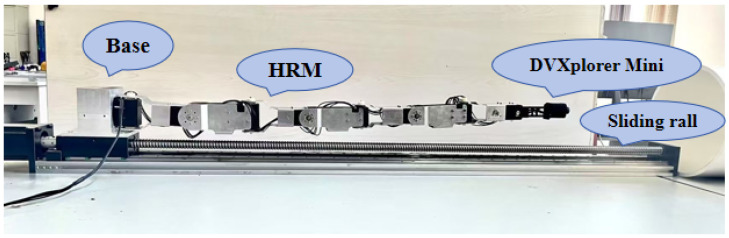
Experimental platform for pipeline defect detection based on an event camera. The setup comprises the key components of the system, including a HRM, a movable base, a sliding rail, an event-based camera, and the pipeline specimen under inspection.

To validate the practicality and feasibility of the proposed pipeline defect detection system, physical experiments were conducted on both steel and acrylic pipelines, as illustrated in Figure 16. In each pipeline environment, the NMPC optimization algorithm was employed to compute collision-free configurations and generate corresponding motion trajectories.

**Figure 17:**
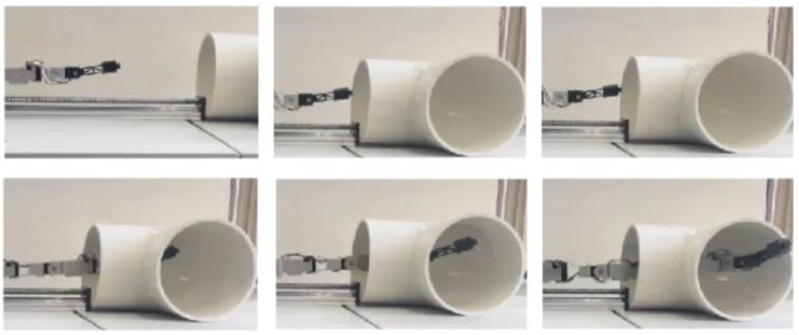
The process of defect detection inside the pipeline using an event camera mounted on the HRM. Six consecutive frames illustrate the collision-free insertion of the HRM into the pipeline. The illuminance at the pipeline entrance is approximately 50 Lux. As the HRM advances deeper into the pipeline, occlusion of the entrance light source by its arm and its own shadow causes the light to diminish, with the minimum illuminance inside the pipeline dropping to approximately 10 Lux.

The generation of the HRM’s collision-free configurations was conducted through MATLAB simulations. Detailed numerical results are presented in Table 9. The table reports the maximum positioning error between the end-effector and the target defect. These metrics are provided for both steel and acrylic pipeline environments. To visually demonstrate the insertion process of the HRM, six consecutive visualization frames are presented in Figure 17. During the pipeline insertion process, the illumination from the pipe entrance to the darkest section ranged between 10 Lux and 50 Lux, corresponding to a low-light environment. Owing to its high dynamic range and asynchronous sensing mechanism, the event camera effectively responded to relative changes in brightness and continuously output an event stream, thereby capturing defect details clearly from the bright pipe entrance to the dim depths of the pipeline. Subsequently, the event camera was inserted into the pipeline by the manipulator to collect data, and the detection results were obtained through ES-YOLO analysis.

**Figure 18:**
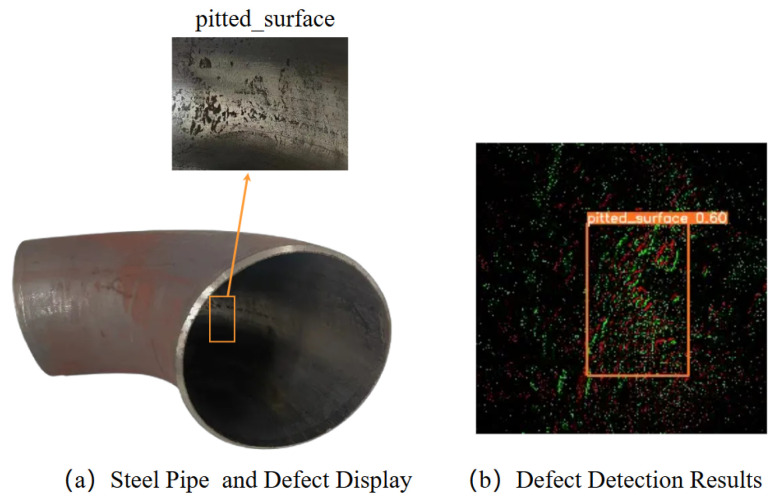
Steel pipe internal defect detection. (**a**) shows the shape and defects of the steel pipe; (**b**) shows the detection results of the steel pipe.

**Figure 19:**
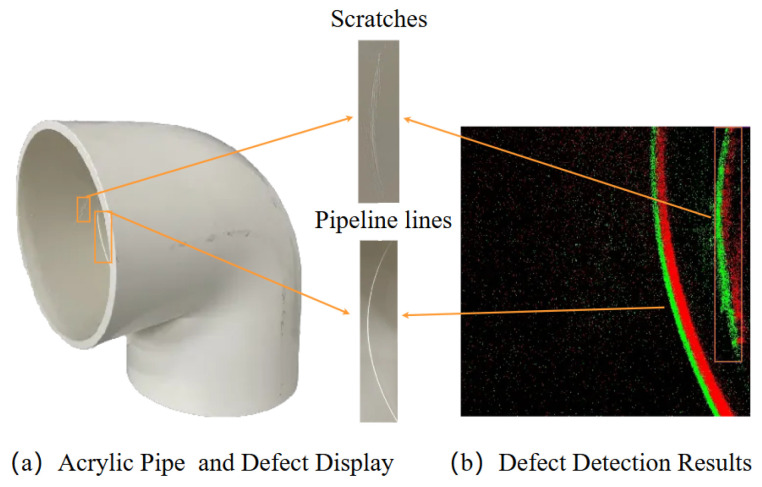
Acrylic pipe internal defect detection. (**a**) shows the structure and defects of the acrylic pipe; (**b**) shows the detection results of the acrylic pipe defects.

Based on the aforementioned experiments, the detection results of the proposed pipeline defect detection system are presented in Figure 18 and Figure 19. The results indicate that the target defects were successfully detected by inputting the pipeline data, collected by the event camera mounted on the HRM, into the ES-YOLO framework. These results demonstrate that the bio-inspired vision-based system could effectively identify defects in pipelines made of different materials.

In summary, the experimental results demonstrate that the ES-YOLO framework exhibited strong applicability in practical pipeline defect detection and possessed cross-material generalization capability, which validates the robustness and effectiveness of the proposed system.

## 9. Discussion

In this study, the ES-YOLO framework successfully detects defects in both steel and acrylic pipes, demonstrating that generating event data through simulation is an efficient and feasible strategy to address dataset scarcity in specialized application domains. This finding establishes a reproducible, data-driven paradigm that can be extended to other industrial and scientific fields lacking native event datasets.

Comparative experiments with traditional RGB vision further highlight the advantages of event cameras in low-light conditions. These results not only confirm their technical feasibility but also open new avenues for robotic applications in visually degraded environments such as unlit pipelines, nighttime inspections, and settings with high-dynamic-range illumination changes. Notably, the system achieves defect detection in near-total darkness, a task that remains challenging for conventional RGB-based methods.

Moreover, the strong robustness exhibited across both steel and acrylic pipelines warrants deeper investigation. We speculate that this cross-material generalization arises from the intrinsic properties of the RGB-to-event conversion process, which primarily encodes brightness changes rather than absolute color or texture information. By filtering out material-dependent surface details while emphasizing geometric contours of defects, this process enhances detection consistency across diverse environments—a property of considerable practical significance for real-world inspection systems.

In this study, the HRM exhibits notable advantages in confined and complex environments that demand high precision and stability. It enables stable, high-precision, non-contact data acquisition and offers a greater payload capacity for carrying advanced sensor suites. Additionally, its energy consumption is significantly lower than that of UAVs. However, the HRM is less suitable for large-scale environments, such as wide-diameter pipelines or oil storage tanks, where UAVs and other mobile robots demonstrate clear advantages. Notably, the ES-YOLO framework is highly portable and can be deployed across various robotic platforms, including UAVs and ground-based mobile robots.

Ultimately, the HRM, UAV, or other robotic systems serve merely as carriers for executing pipeline inspection tasks. Deploying the ES-YOLO framework on different platforms to support diverse engineering applications presents broad prospects for practical deployment.

However, the current approach has a fundamental limitation: to leverage the mature YOLOv8 architecture, the asynchronous event stream must first be converted into discrete event frames before being input into the ES-YOLO framework. While this conversion step facilitates the application of advanced detection algorithms, it compromises the intrinsic advantages of event cameras, such as microsecond-level temporal resolution and low latency. Therefore, future research should prioritize overcoming this bottleneck by exploring advanced algorithms capable of directly processing raw event streams. A particularly promising direction is the use of Spiking Neural Networks (SNNs), whose event-driven and asynchronous processing characteristics are inherently compatible with the data format of event cameras, offering the potential for low-latency and low-power defect detection. In addition, asynchronous convolutional networks and graph-based methods that operate directly on event streams also warrant further investigation. Ultimately, the goal is to deeply integrate these emerging technologies with the HRM detection system to enhance the generalizability and practical utility of the entire framework.

## 10. Conclusions

This article proposed an ES-YOLO framework to enhance defect detection in low-light pipeline environments, which leveraged an event camera based on bionic visual perception. To address the issue of scarce event datasets, the RGB NEU-DET dataset was converted into the N-neudet event-based dataset using the ESIM module. Subsequently, the pipeline defect detection model was trained on the generated event data via YOLOv8s. To verify the accuracy of this framework in low-light environments, two sets of comparative experiments were conducted to assess the defect detection performance of RGB cameras versus event cameras within both steel and acrylic pipelines. The results showed that RGB cameras fail to effectively identify defects, while event cameras demonstrated clear advantages under low-light conditions. Furthermore, the pipeline defect detection system based on a snake-like HRM was developed, and its feasibility and detection accuracy were validated through physical experiments in realistic pipeline inspection scenarios.

Future work will focus on expanding the breadth and depth of application for the proposed HRM-based detection method. To achieve real-time and efficient system operation, low-power models such as Spiking Neural Networks (SNNs) will be deployed on embedded hardware to reduce inference time and improve energy efficiency. At the system architecture level, migration to the ROS 2 framework will be explored to enhance modularity, stability, and scalability. On the perception level, efforts will target key challenges such as real-time 3D modeling and dynamic path planning in unknown environments—both critical to improving system autonomy. To validate and improve the framework’s generalizability, its application will be extended beyond pipeline interiors to encompass a broader range of industrial geometries, including ventilation ducts, mining tunnels, and confined industrial cavities. The ultimate goal is to develop an automated and intelligent inspection system capable of adapting to diverse environments while maintaining high autonomy, efficiency, and robustness for inspection tasks in various constrained spaces.

## Figures and Tables

**Table 1:** Training parameters.

Parameter	LR	Batch	Image	Monmentum	Epoch
Value	0.01	64	640*640	0.937	100

**Table 2:** ES-YOLO validating results.

Class	P	R	mAP@0.5	FNR	FDR
All	0.952	0.657	0.801	0.343	0.048
Crazing	0.817	0.0714	0.238	0.9286	0.183
Inclusion	0.957	1	0.995	0	0.043
Patches	0.982	0.75	0.971	0.25	0.018
Pitted	1	0.62	0.876	0.38	0
Rolled	0.956	0.5	0.732	0.5	0.044
Scratches	0.998	1	0.995	0	0.002

**Table 3:** Comparison of training results between RGB and event dataset.

Framework	Dataset	P	R	mAP@0.5	Inference Time
ES-YOLO	N-neudet	0.952	0.657	0.801	79.167 ms
YOLO	NEU-DET	0.84	0.637	0.773	69.444 ms

**Table 4:** Comparison results of event camera and RGB camera detection in steel pipes.

Target Defect	A	B	C
**Illuminance**	45 Lux	13 Lux	2 Lux
**Event camera $D_{r}$**	94%	90%	84%
**RGB camera $D_{r}$**	66%	30%	10%

**Table 5:** Comparison results of event camera and RGB camera detection in acrylic pipes.

Target Defect	A	B	C
**Illuminance**	45 Lux	13 Lux	2 Lux
**Event camera $D_{r}$**	82%	76%	68%
**RGB camera $D_{r}$**	34%	10%	0%

**Table 6:** Composition of the pipeline defect detection system.

Component	Specifications
HRM	Three high-torque XH540-W270-R motors
	Five lightweight XH430-W270-R motors
The sliding rail	Stepper motor
	Lead screw
	Base
Controller	PC(Intel(R) Core(TM) i5-8265U CPU @ 1.60GHz)
Protocol	RS485 protocol to control HRM
	RS485 protocol to control sliding rail
Event camera	DVXplorer Mini

**Table 7:** D-H parameters of the snake manipulator.

Link *i*	$\theta_{i}\;{(}^{\circ })$	$\alpha_{i}\;{(}^{\circ })$	$a_{i}\;\left(m\right)$	$d_{i}\;\left(m\right)$
i=1,3,5,7	$\theta_{i}$	$-90^{\circ }$	0.1	0
i=2,4,6,8	$\theta_{i}$	$90^{\circ }$	0.1	0

**Table 8:** Minimum safe passage radius of the HRM for different pipeline curvatures.

Curvatures	$D_{\mathrm{safe}}$	$D_{\max}$	Minimum Pipeline Radius for Safe Passage
$45^{\circ }$	4 cm	2.53 cm	6.53 cm
$90^{\circ }$	4 cm	3.72 cm	7.72 cm
$135^{\circ }$	4 cm	5.13 cm	9.13 cm

**Table 9:** Geometries of the two pipelines and experimental error results.

Pipe Type	Radius	Pipe Angle	Maximum Error
Steel	8 cm	$90^{\circ }$	1.542 cm
Acrylic	10 cm	$90^{\circ }$	1.947 cm

## Data Availability

The original contributions presented in this study are included in the article.

## Abbreviations

The following abbreviations are used in this manuscript:

HRM	Hyper-Redundant Manipulator
NMPC	Nonlinear Model Predictive Control
SCA	Snake-inspired Crawling Algorithm
DOF	Degrees Of Freedom
D-H	Denavit–Hartenberg
